# Harnessing Globalization: An Everlasting Challenge

**DOI:** 10.1007/978-3-030-55400-2_1

**Published:** 2020-10-17

**Authors:** Tarja Halonen

**Affiliations:** 1 Office of the President of the Republic of Finland, Helsinki, Finland; 2 Faculty of Law, University of Helsinki, Helsinki, Finland; Office of the President of the Republic of Finland, Helsinki, Finland

## Abstract

In this chapter, President Tarja Halonen reflects on her experience serving as co-chair of the ILO World Commission on the Social Dimension of Globalization from 2002 to 2004. The World Commission’s work in achieving fair and inclusive globalization, and promoting decent work are discussed in detail. Major challenges to and prospects of the ILO as an international organization are reflected even more broadly, to build a view of the future of labour.

## Introduction

At the turn of the century, globalization was a hot topic. Many took part in demonstrations for—or against—it. For some, globalization was a logical continuance of increasing production efficiency and of a global division of labour. Others felt that globalization only served the needs of capital and production and ignored people and their needs.

The ILO World Commission on the Social Dimension of Globalization reached a consensus to add to the UN Millennium Goals the concept of “decent work”, which has been part of the Sustainable Development Goals from the start. Balanced development requires the inclusion of people and human needs.

How did it all happen? This is how I, as someone who was involved in the process, understand and remember it. This is not an academic study or a personal memoir but rather my personal perspective on what factors and circumstances led to the emergence of a new attitude towards globalization and, moving forward, to the emergence of the idea of sustainable development. This is also a statement about the benefits and risks of globalization in the contemporary world and in these coronavirus-ridden times.^1^

## The Historical Conflict Between Capital and Labour

### The Trade Union Movement as a Proponent of Employee Benefits

The history of the trade union movement is different from country to country, but usually it has been part of or occurred in parallel with a political labour movement. Therefore, it shares the ideals of democracy, human rights and rule of law. Similarly, from early on, the trade union movement has valued international collaboration. Technological development led to the emergence of/need for industrial labour; the trade union movement arose from the need to address poor working conditions.

This was the fundamental nature of the trade union movement at the time the ILO World Commission was established, and indeed remains so to this day. When work and working environments change, new challenges are posed to employees and thereby to the trade union movement. Certain problems cannot be solved in negotiations between employer and employee, but require broader, structural solutions. This calls for extensive collaboration across different areas of society.

The World Commission also adopted this broad-based approach. Its proposals extend beyond the original focus of work-related issues. This was deliberate. Juan Somavia, then Director-General of the ILO, was closely connected to the World Commission, and shared our thinking,^2^ through the founding letter of the World Commission as well as through the selection of its members.

My co-chair, Benjamin William Mkapa, and I strove to paint an optimistic picture of opportunities for change, even though we did not have a mandate or even a desire to present a proposal outside the ILO. However, I knew, of course, that the issue had been broadly discussed within the UN family. The hopes and desires for change turned out to be tremendous. This is how we, the co-chairs of the ILO World Commission, phrased it: “Our proposals are ambitious but feasible. We are certain that a better world is possible.”^3^

### Controversial Tripartism

It is probably worthwhile to underline that this awakening came early, compared to many other national developments in the history of political and professional labour movements.

When the ILO was established in 1919, the rights of workers and their structurally weaker contractual status were on the table. Trade unions were the natural answer. However, it was also clear that improving the status of employees is only possible in a democratic state that recognises the equal rights of all individuals. Rule of law and good governance also protect the status of workers. A state has a general obligation to protect its people, and therefore all employees.

Recently, the triangle—employers, employees, the government—as contractual parties has gained less attention. However, in exceptional times, employers also seem to be more willing to adopt a more positive approach to the tripartite system. The trade union movement is needed to bring a nation together. Tripartism was also a central philosophy in the rebuilding of Europe after the Second World War and during the financial crises early in the millennium. Currently, we are seeing signs of this during the coronavirus epidemic of 2020.

Throughout the decades, the ILO has shown interest in tripartism to a varying degree. It gained increasing importance in the 1960s and came back into focus at the turn of the millennium. Strong nation states are also needed to keep globalization in check.

## Global Responsibility and Solidarity

The World Commission was not operating alone or in a void. For many, the Millennium Development Goals, set by the UN in 2000, represented the first statement in favour of global social justice.^4^ The Goals are not a legally binding commitment but, rather, a political commitment. The member states took them quite seriously, though. Developing countries in particular considered them a major win. They could have been implemented more quickly but the UN monitored them closely and addressed some of their shortcomings.

I followed this process closely, because the Millennium Summit of September 2000 was jointly presided over by Finland and the exiting Presidency of the UN General Assembly, Namibia. My country was about to take over. It is customary in the UN that important summits are co-chaired by representatives of North and South, as was the case in 2000.

I had been elected President of Finland in our March elections, and therefore co-chaired the historic meeting with President Sam Nujoma of Namibia. Former Prime Minister Harri Holkeri assumed the role of President of the United Nations General Assembly during Finland’s one-year presidency. I knew him well. When I was a minister for the first time, it was in the Holkeri government of the 1980s. Looking back, the Millennium Development Goals were a great achievement. Poverty—extreme poverty in particular—declined. Disparities of wealth continued to grow, however.

Progress was made in healthcare: the number of women and children dying during childbirth shrank significantly, and the infant mortality rate declined dramatically. Girls’ and women’s health still lagged behind that of men and boys. Many more girls and boys gained access to primary education, but the quality of education failed to meet expectations. Girls’ education was too often interrupted by family obligations.

Plans were made to address social injustice through development cooperation. In the Monterrey Consensus, the countries involved endorsed giving at least 0.7% of their GNI in official development assistance, in which education and healthcare would play a central role in tackling extreme poverty and hunger. Many felt an urgency to remove injustice from the prevailing economic system. The role of women needed to be improved.^5^

The key message of the Millennium Development Goals was global solidarity; yet, they did not mention the world’s most common source of income: work. There was also no reference to social security. From this point of view, too, the Report of the World Commission on the Social Dimension of Globalization was a welcome addition.

## Globalization: A Multifaceted Challenge and an Opportunity

### Globalization Changed the Way We Work

The emergence of an international division of labour was a lengthy process. At the turn of the millennium, different parts of the world witnessed it differently. Generally speaking, there was a division between industrial countries and developing countries—between North and South. A closer look showed that there were winners and losers in both groups. There were also winners and losers within individual countries.

In Europe and North America, globalization was largely considered to involve a division of labour related to increasing production efficiency. The invention and use of new technology after the Second World War strengthened opportunities—and the need—for an international division of labour. In a way, information technology shrank the world. But what makes production more efficient does not necessarily benefit the worker.

Globalization did, however, give poor countries a new opportunity to narrow down the income gap and not just to the traditionally poorer countries of the global South but also to industrial countries with a smaller economy—such as my own country, Finland. We remained a well-educated and agile country that was able to create such phenomena as Nokia. It can be said that Finland’s Linus Torvalds created an operating system that now runs a large part of the internet. Globalization appears to provide unprecedented opportunities for economic growth, not to mention improved health and wellbeing.

In Africa, Asia and Latin America, globalization is typically considered part of the same continuum as colonialism. The opinions of the members of the World Commission reflected these diverse views. The Millennium Development Goals were seen as the beginning of global compensation programmes. The Monterrey Financing for Development conference, whose goal was to increase official development assistance from 0.7% of GNI, had boosted faith in the changes under way. The Asian members of the World Commission were more optimistic than other developing countries about being able to close the divide between them and European countries and the USA by means of globalization. Upon closer inspection, we see that most new investments were made in the two largest Asian countries: China and India.

What the workers and labour representatives of different countries had in common was a concern that people and their needs would be sidelined by globalization. Demonstrations against globalization grew larger. People opposed the exportation of jobs to developing countries. Multinational enterprises in particular were seen as the culprits. At the same time, governments were blamed for not protecting their citizens against unemployment. The international finance world was blamed for supporting large corporations.

In a market economy, governments had limited possibilities to protect jobs, however. They mostly encouraged companies to retrain former employees who had been left jobless because of globalization. However, the training and reassignment of unemployed people was insufficient. Those with less education lost jobs, and any new jobs were given to younger people with a higher education. In the “old” industrial countries, too, society became polarised into proponents of and opponents to globalization.

Let’s take a practical example:

In February 1997, the French car manufacturer Renault decided to close its plant in Vilvoorde, Belgium. After making the decision to cease manufacturing, Renault announced it publicly without first informing or consulting its European Works Council. The announcement was followed by demonstrations and a strike that took place simultaneously in Belgium, France and Spain, of which the latter was to be the new home of the former Vilvoorde operations. The case was brought to court in both Belgium and France. This did not stop Renault from closing down its Vilvoorde plant, and most of the employees lost their jobs.^6^

The European Union, alongside many other international organisations and governments, still had faith in managed globalization.

Thus, in a way, the tripartite principle behind establishment of the World Commission on the Social Dimension of Globalization was a natural consequence in addressing the polarising political situation at the time.

### Multinational Corporations: Aspirations of Autonomy

Multinational corporations grew rapidly. Their size was a challenge not just for the trade union movement but also for governments. They adopted a discourse according to which multinational corporations had no home base. They seemed to want to form an independent community of their own, outside the jurisdiction of any country. The role of nation states as defenders of their people became weaker.

At the same time, large corporations in particular started to streamline operations by outsourcing non-core functions, such as cleaning and transportation, to formally independent operators. In reality, these subcontractors did not become independent entrepreneurs but subcontractors who were completely dependent on the head company. Their status was similar to that of employees, but health and safety rules and social security did not apply to them.

Furthermore, particularly in developing countries, many workers were excluded from employment calculations. Many agricultural workers belonged to this group. They did not own the land they were cultivating, yet they were not counted among the employees of the landowner. Most of these workers came from developing countries but, because of their status, they were not eligible to take part in any of the training or support programmes provided in the development cooperation agreement. Thus, these people were not covered by employee health and safety or job security schemes, nor were they eligible to receive entrepreneur benefits.

In many developing countries, the share of such informal work was, and still is, significant. There are many reasons for this phenomenon: the remnants of colonialism, the legal underdevelopment of ownership, women’s limited rights to own land, and so forth. Informal work was keeping people alive but at great risk.

These are the reasons why we in the World Commission considered it absolutely necessary to address both the issues and the solutions much more broadly than labour legislation would have permitted. We considered it a political rather than a legal question.

### A Polarised World

The world had become polarised in many ways. The UN Millennium Development Goals set at the turn of the millennium remain founded on global solidarity and justice. They can also be seen as an attempt to mitigate the divide created by globalization between the wealthy global North and the poor South. They did not, however, highlight the fact that poverty could also be found in the industrial North. Unemployment was rising, and the standard of living was declining in many previously wealthy areas. People lost faith in the future. While awareness of the help needed in the poor global South was increasing, unemployed industrial workers did not consider themselves oppressors of the South. A breeding ground for political extremism and populism was created.

The international sentiment was already getting bleaker. On 11 September 2001, Al Qaida destroyed the twin towers of the World Trade Center. Hundreds of people died in a matter of minutes. The USA declared war on terrorism. Fear and deep mistrust spread across the world. It seemed unlikely that any new international treaties could be concluded.

The Millennium Development Goals were a diplomatic surprise of sorts. They raised important issues ranging from poverty to health and education. They did not, however, mention work as a source of wellbeing. The Millennium Declaration was agreed upon at the UN relatively quickly but resources for its implementation were lacking. Concrete follow-up was also needed.

### The World Commission Was Also Welcomed by the UN Family

In this polarised situation, the ILO decided to establish a World Commission to investigate the social dimension of globalization.^7^ It was a rather bold attempt to bring a highly split group—all with good arguments—to the same table. The World Commission, set up in 2002, was welcomed into the UN family: not to the UN itself but to the ILO, its agency organisation in Geneva.

The chairs and members of the World Commission formed a diverse group from around the world. The aim was to guarantee that the World Commission would have broad-based expertise on the many aspects of globalization. The members represented employers, trade unions, governments, academia and civil society, including the women’s rights and indigenous peoples’ movements.

Its diversity was both a blessing and a challenge. We spent a significant share of the relatively limited time we were given in building trust between different parties. This so-called honeymoon period was relatively successful. In my opinion, everyone was interested in each other’s perspectives. Where else could the CEO of a large Japanese corporation have had reason or the time to learn about the experiences of a South African freedom fighter, or a European statesman ask a nurse working with the indigenous peoples of the Philippines for their opinion on how globalization was affecting people’s living conditions?

More attention was paid to differences between the lives of women and men, but it remained a minor theme in the talks, despite the participation of many active women and informed men. The strong and vivid presentations given by Ruth Cardoso and Hernando de Soto from Latin America have stuck in my mind. Gender equality issues were being raised across the world, but this was less obvious in the World Commission than in some other UN events. In the final version of the text of the Commission report, however, equality featured quite strongly.

We were lucky to have an excellent secretariat that had already worked on defining our scope within the ILO. The members of the World Commission included economic policy experts such as Joseph Stiglitz and Hernando de Soto, who, together with Giuliano Amato, the former prime minister of Italy, and German politician Ernst Ulrich von Weizsäcker, joined forces to take an in-depth look at the relationship between economic development and democracy.

China was represented by Lu Mai who comes from a research institute and was a very active participant. We also visited China. The World Commission marked the beginning of long-term collaboration between myself and several other members, including Valentina Matvienko from Russia, who was then the Mayor of St. Petersburg. The members of the World Commission were not only top experts but also charming personalities.

I had not met my co-chair, Benjamin William Mkapa from Tanzania, before. After spending hours with him and the Secretariat planning future action and discussing what might be realistic steps to take, we became close. Some of the members of the World Commission were well-known and had long careers, while for others, their careers were just about to take off. All in all, I would like to say that cooperation between the members of the World Commission left me with an excellent impression of competence and cooperation, which I benefited from on many later occasions. The ILO’s Director-General, Juan Somavia, had built a solid foundation.

Once, at a World Commission summit, I attempted to sum up the different viewpoints of the participants. I likened globalization to a train that runs on its own tracks, fuelled by its own power. Industrial countries were seated in first class, having purchased tickets with other people’s money. Asians were sitting on hard seats in third class. And Africans were still waiting at the station, wondering whether they should get on the train or not. Victoria Tauli-Corpuz, who represented the indigenous peoples of the Philippines, said it seemed to her that the train was running on their land without permission, while they just kept trying to avoid getting run over by it.

## Proposals of the World Commission

The practical near-future objective of the World Commission was to add decent work and employment to the ILO principles, the UN Millennium Development Goals and, possibly, the goals of other international organisations. The same aim was also to be included in government programmes.

The World Commission’s suggestions for fair globalization involved several levels. The most comprehensive aim was to change the fundamental nature of globalization: to move away from narrow, production-centric thinking towards a broad-based approach attentive to the needs of the people. In addition to the economy, democracy, human rights and good governance must be promoted.

Corporations and other economic actors must abide by these rules just like everyone else. The most radical idea of the World Commission was to incorporate employment and the democratisation of labour into a more socially just world. We were of the opinion that globalization had taken a wrong turn because it materialised people. People want to earn a living and social independence through working: doing something that others consider to have exchange value. Thus, work is important not only because it provides a livelihood but because it integrates people in society. To put it conversely, working life is part of a democratic society where all the same laws apply as elsewhere in society.

Respect for human rights in working life was an integral element of ILO core labour standards.^8^ This means that forced and child labour are banned. All people must have the same human rights, including freedom of expression and freedom of association. Workers must have the same human rights inside and outside their workplaces. Thus, an employer’s power over an employee is not unlimited. The World Commission viewed core labour standards and respect therefor as important. Moreover, social dialogue is needed. The right to organise and collective bargaining are core elements of social dialogue.

I have sometimes been asked why we chose the phrase “decent work”. We did not want to use the word “good” because of its connotations. The terms “good job” and “good life” did not convey what we wanted. Decent means that we are not quite there yet but very close; and “decent” is also a synonym for “fair”. It was a realistic objective that we could build on. It is a global demand. It must be true everywhere. I remember the ILO Secretary-General, Juan Somavia, thinking along the same lines even before that. However, I was not involved in the process at that point.

The work of the World Commission was marked by an optimistic view of people’s ability to control the world and globalization. We believed that globalization would increase the efficiency of production, benefit individuals and societies alike and integrate humanity in a way that would make conflicts and wars less likely.

## Globalization Management Starts with a Strong State and Continues with International Collaboration

Many globalization scholars have theorised that nation states would become less important as globalization gains strength. This would probably be the case if the market economy had developed freely under globalization. Such a development would probably not attend to human needs and desires.

The World Commission had faith in the nation state because, at the moment, the state is the largest unit that people can impact. Therefore—without underestimating international organisations—we should try to make states stronger, instead of wishing them to grow weaker. They still constitute the best protection for the people. However, citizens should be more aware of what their representatives do in international forums.

In addition to globalization itself, the World Commission wanted to draw strong focus on attempts by financial market operators to impact the way countries can control the detrimental effects of globalization. For example, developing countries were pressured to allow movement of investments and capital as freely as possible, even though it was known that the rapid liberalisation of finance had proved highly destabilising to economic policymaking in a number of nations from Latin America to East Asia. The report of the World Commission states that developing countries should be allowed to take a cautious approach to freeing up movements of capital.^9^ World trade rules hindered this type of selective protection or strategic promotion of domestic industries that played a part in the economic development of successful industrialised countries in Europe, North America and Asia. Some of these proposals were adopted.

Thus, international organisations should democratise their own rules and improve the efficiency of their own operations and mutual cooperation. The UN reform has been ongoing during the term of every UN Secretary-General of this millennium. It takes a long time. There should also be closer cooperation between the specialised agencies of the UN. According to the World Commission, special attention must be paid to coordination or collaboration between the UN and organisations close to it, such as Bretton Woods institutions.^10^

The international community continued to have the same kind of optimism as when the Millennium Development Goals were set. They believed that comprehensive management of global economic policy could be achieved, even though negative phenomena such as the accumulation of wealth, polarisation and the crisis of financial capitalism were present. The World Commission’s idea of a comprehensive and multipolar but controllable globalization lives on.

The Millennium Development Goals made few references to the environment. The report of the World Commission did not highlight environmental impacts in any major way. It was not until the Sustainable Development Goals—the Rio Summit^11^ and Agenda 2030^12^—that nature and the environment, gender equality and the status of women were brought to the fore.

## Did the World Commission’s Objectives Change the World?

The report of the World Commission sparked discussion around the world.^13^ Even during the working stage, the Commission made its work visible not only through its diverse membership but also through its meetings and visits around the world. This was effective, albeit arduous.

The ILO approved the recommendations of the report, which was submitted to its Governing Body and the International Labour Conference (ILC) in 2004.^14^ Also the Director-General of the ILO submitted his proposals to the ILC for a strategic response to the World Commission’s recommendations, covering some of the key fields of work of the ILO: national policies to address globalization, decent work in the global production system, growth, investment and employment, a socio-economic floor, international migration, the international labour standard system, and the role of tripartism.^15^

Today, fair globalisation is one of the mainstream strategies of the ILO addressed in its programmes across the world.^16^

Decent work was added to the Millenium Development Goals in 2005.^17^ It has been part of the Sustainable Development Goals from the start.

Decent work has featured widely in different UN programmes.^18^ I have myself twice been part of UNCTAD reform processes. In addition, the IMF has, to some extent, included the recommendations of the World Commission in its policies.^19^

## Epilogue or: What’s Next, Globalization?

Globalization evolved into an effort to increase production efficiency. It was the next step of internationalization and the international division of labour. Without a doubt, globalization has in many ways been a great economic success; it has also played a part in tackling poverty. During the course of its evolution, globalization has become more cost efficient but also increasingly vulnerable. Furthermore, it has not proven to be as independent or omnipotent as its most fervent fans have led us to believe, because even a multinational corporation operates on national territory or territories. Nation states tend not be controlled by individual companies, even though the financial—and thereby political—influence of multinational corporations has greatly increased. It takes time to worm one’s way into a societal power structure. To assume political power, multinational companies can use semi-democratic means and pressure decision-makers to give them tax benefits by arguing that these would boost employment or otherwise benefit voters. We have learned from court cases around the world that heads of state, government leaders and officials sometimes take bribes.

The free movement of goods and services is a prerequisite for free trade. Multinational companies would not be able to operate without bilateral or multilateral cooperation between governments. In most nation states, citizens have some influence over the choice of the country’s decision-makers. This requires that they believe that they can benefit from the issues that their elected leader or leaders are lobbying for. This should also be the case with free trade and globalization.

Globalization will not disappear, and nor will nation states. Therefore, their mutual relationship must be rebuilt in a more sustainable way. A more humane but also economically more efficient form of globalization is only possible with the support of strong and democratic nation states that are open to international collaboration.

People desire financial wellbeing and social justice; that is the kind of development they are looking for. At the same time as the ILO World Commission was working on its report, the UN Millennium Development Goals were already being implemented. The action programme, which emphasised social justice, was launched in 2004. In 2015, the Sustainable Development Goals were approved and they contain the same principles as the programme, with an added demand for environmental welfare.^20^ The link between these factors is logical and straightforward but, with so many of them to address, implementing changes is increasingly difficult. Furthermore, employment has been awarded relatively little attention in the programme-setting for sustainable development even though unemployment or fears of unemployment are among the key reasons behind the rise of populism.

The report of the ILO World Commission on the Social Dimension of Globalization covers a lot more than just decent work and employment. Globalization as a philosophy is in need of comprehensive reform. Sustainable development is founded on human rights, democracy and rule of law: they build trust between people. This trust must extend to globalization. The World Bank in particular along with the IMF appears to have come to realise the significance of rule-based international collaboration. As early as the financial crisis of 2008, governments were forced to pause and consider the warning given by the World Commission regarding the risks of unregulated international finance capital.

The problems that the European Union has faced with the single European currency have starkly demonstrated the significance of collaboration and honesty—or rather the consequences of a lack thereof. Member States have committed to a single currency but hesitate to observe a common monetary and financial policy without legally binding rules. This is one of the topics addressed by the Nobel-prize winning Joseph Stiglitz in this volume.^21^

The direction in which the WTO has developed in the 2000s is a stark reminder of the fundamental problem related to globalization, or rather the market economy. Even if capital and production dictated these rules, any mutually-agreed rules aiming to promote change would be overrun by a struggle for the survival of the fittest. The World Trade Organization getting stuck in court is a great example:

The crisis of the WTO’s dispute settlement system began on 10 December 2019, when the term of two of the remaining three Appellate Body members expired. As the United States refused to initiate the selection process and appoint new members to the organization’s Appellate Body, the Members were unable to reach a consensus to fill the vacant positions.^22^ With only one member in office, the Appellate Body has become dysfunctional and is unable to hear new appeals. The blockade of the Appellate Body has paralyzed the two-tiered dispute settlement system in the WTO. Despite the members’ attempts to restore a fully functioning Appellate Body, the impasse remained unresolved.^23^

### The Future of Labour

The world and, with it, the world of labour have changed rapidly since the report of the ILO World Commission on the Social Dimension of Globalization was published. The report of the ILO Global Commission on the Future of Work, titled “Work for a brighter future”, is a statement on this topic. It does not deny the future existence of globalization but requires that social justice be observed in its implementation.

Furthermore, people want to feel useful. Therefore, demands for livelihood or, for example, a universal basic income—which has gained popularity in the Nordic countries in particular—were not the only focus; rather, the significance of people was. Work is gaining increasing importance. For example, youth surveys from Finland point to a similar trend, showing that young people want to do work that matters and that they are not merely motivated by the opportunity to earn money.

In order to make this dream a reality, the ILO Global Commission on the Future of Work suggests that we invest in people not only through education but also through work and work-related institutions. In other words, labour must have an economic, humane and environmental impact. Future society will be increasingly information-based, and this requires the creation of opportunities for life-long learning.

### What Can We Learn from the Coronavirus (COVID-19) Epidemic?

As I am writing this in April 2020, we do not yet know what returning to so-called “normal” will look like once the epidemic is over. However, we already know that this epidemic has had an unexpectedly significant impact on people’s health and wellbeing.

Tackling the coronavirus has required both a national and an international effort. Currently, the infection rate is approximately 3.1 million, and the death toll is roughly 223 thousand.^24^ The virus does not recognise national or other borders. For comparison, a total of 2.7 million people die from work-related reasons each year.

This tiny little virus turned our world upside down in what seemed like seconds: it emptied our calendars, closed our borders and made us change our behaviour both voluntarily and by coercion.

In addition to human suffering, the corona epidemic sparked a dire economic downturn. We are yet to learn its scope. The virus also forced people to consider the production-related, economic and humane consequences of globalization.

We realised how dependent we are on each other. We missed our families and friends in different ways—but we also missed being able to simply “be” safely around other people at school, at work, and in cafes, shops and parks. In other words, being human amongst other humans.

If there is a lesson to learn from COVID19 it is that our world is highly fragile.

The ways in which globalization developed, or was developed, resulted in a gigantic but extremely vulnerable system. An increasing number of people have come to observe how fragile the foundation of globalization is: a system driven by profit-making alone is simply not compatible with sustainable development. Economic production systems must be able to operate under common rules, not outside them.

I am an optimist. I believe that we want to change the world and that we will indeed change the world.

## Footnotes

1 This article is a translation from Finnish into English.

2 ILO (1999).

3 World Commission on the Social Dimension of Globalization (2004), p. ix.

4 Resolution adopted by the UN General Assembly: United Nations Millennium Declaration (A/RES/55/2).

5 UN (2003).

6 For a discussion of the many legal dimensions and legal procedures related to the case, see Liukkunen (2005), p. 20.

7 ILO (2004a).

8 See ILO, Declaration on Fundamental Principles and Rights at Work. Adopted by the International Labour Conference, 86th Session, Geneva, 18 June 1998 (Annex revised 15 June 2010). The Declaration underlines four core labour standards, namely freedom of association and the effective recognition of the right to collective bargaining, the elimination of all forms of forced or compulsory labour, the effective abolition of child labour, and the elimination of discrimination in respect of employment and occupation.

9 World Commission on the Social Dimension of Globalization (2004), pp. 90 and 114.

10 Ibid., pp. 113–114.

11 See Resolution adopted by the UN General Assembly on 27 July 2012: The future we want (A/RES/66/288).

12 See Resolution adopted by the UN General Assembly on 25 September 2015: Transforming our world: the 2030 Agenda for Sustainable Development (A/RES/70/1).

13 ILO (n.d.-a), ILO (2004b); Resolution adopted by the General Assembly on 2 December 2004: A fair globalization: creating opportunities for all – report of the World Commission on the Social Dimension of Globalization (A/RES/59/57).

14 ILO (2004c).

15 ILO (2004d).

16 See, for example, ILO (n.d.-b, n.d.-c). See also ILO Declaration on Social Justice for a Fair Globalization. Adopted by the International Labour Conference, 97th Session, Geneva, 10 June 2008.

17 Resolution adopted by the UN General Assembly on 16 September 2005: 2005 World Summit outcome (A/RES/60/1), para 47.

18 See, for example, UN Women (n.d.) and UNDP (n.d.).

19 Jenkins et al. (2007), paras 89 and 97.

20 Resolution adopted by the UN General Assembly on 25 September 2015: Transforming our world: the 2030 Agenda for Sustainable Development (A/RES/70/1).

21 See Stiglitz (2020).

22 See WTO (2020) Minutes of Meeting of the General Council, Geneva, 9–10 December 2019, pp. 12–33.

23 See WTO (2020) Minutes of Meeting of the Dispute Settlement Body, Geneva, 27 January 2020, pp. 14–19.

24 WHO (2020).
